# Mesoamerican *Cypripedium*: Mycorrhizal Contributions to Promote Their Conservation as Critically Endangered Species

**DOI:** 10.3390/plants11121554

**Published:** 2022-06-12

**Authors:** Mauricio Moreno-Camarena, María Pilar Ortega-Larrocea

**Affiliations:** 1 Posgrado en Ciencias Biológicas, Universidad Nacional Autónoma de México, Mexico City 04510, Mexico; novaorchidcc@gmail.com; 2 Departamento de Ciencias Ambientales y del Suelo, Instituto de Geología, Universidad Nacional Autónoma de México, Mexico City 04510, Mexico

**Keywords:** habitat destruction, in vitro germination, orchid conservation, orchid mycorrhiza

## Abstract

In the valuable orchid genus *Cypripedium*, the section Irapeana consists of a distinctive group of Mesoamerican species that is formed by *Cypripedium dickinsonianum* Hágsater, *C. irapeanum* Lex., and *C. molle* Lindl. All lady slipper orchids exhibit different distributions and abundances. Data analysis that used herbarium accessions and field investigations indicated that the habitats of these three species have been dramatically reduced. Prospecting for suitable habitats based on climatic, vegetation, and soil parameters allows us to predict potential distributions. Conservation strategies, such as ex situ propagation by asymbiotic and symbiotic approaches, have indicated that the culture media used are a determining factor for seedling development. Mycorrhizal isolates play a main role in the compatibility and further development of germinated seeds. The fungi isolated from adult plants belong to two different families, which makes it possible that widely distributed *C. irapeanum* populations will be fungal-specific as well as restricted for *C. molle*. Root mycorrhization patterns occur high on the secondary roots. In contrast with other species of the genus, in situ germination can occur over a short period of two months, but we have documented periods as long as ten years. *Cypripedium* is a highly problematic genus for ex situ conservation because the germination requirements and cultures are poorly documented, and there is great urgency for in situ conservation to develop strategies for identifying hotspot habitats and actualize the protection status to avoid extinction of this genus.

## 1. Introduction

The terrestrial genus *Cypripedium* is one of the most appreciated in the orchid family and is found in the natural environment, botanical gardens, natural parks, and scientific and private collections [1,2]. The origin of the name comes from the Greek root *Cypris* that refers to Aphrodite’s sandal pedilon because of the globose-sac-shaped flower lips. Along with modifications of sepals and petals, columns with two anthers and the presence of staminodes make them unique relative to all other orchids. The genus contains approximately 50 species that are distributed in the Northern Hemisphere in mountain woodlands, grasslands, shrubs, or swamps, and is mainly associated with *Quercus* or *Pinus* forests [2,3,4,5,6,7,8,9]. All species are endangered by overcollection, anthropogenic activities, and climate change [10,11].

**Figure 1:**
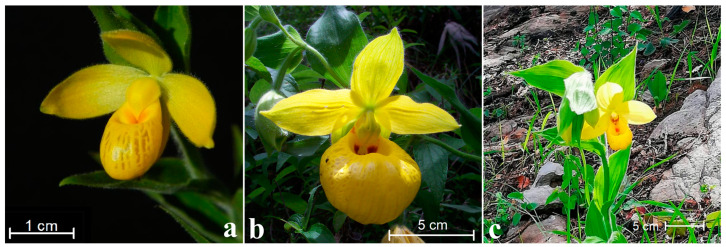
Mesoamerican *Cypripedium*: (**a**) *C. dickinsonianum*, (**b**) *C. irapeanum* and (**c**) *C. molle*, pictures taken by Javier Fortanelli, Mauricio Moreno, and Octavio Gabriel in the years 2014, 2010 and 2019, respectively.

Mesoamerica has been postulated to be the origin center of the genus *Cypripedium* with the section Irapeana, which is a sister clade to the other members of this taxa [2,4,7,12,13,14]. The section contains three species, namely, *C. dickinsonianum* Hágsater, *C. irapeanum* Lex., and *C. molle* Lindl. [8]. *C. irapeanum* from Irapeo, which is located in Michoacán State of Mexico, is the type species, but the type locality no longer exists [13,14,15]. There is remarkable morphological variability among *C. irapeanum* populations, which are likely to consist of a complex of species to be resolved by using molecular markers [16]. Morphological variations, mainly in size, are also observed in *C. dickinsonianum* populations from the northeastern Sierra Madre compared to those in the Chiapas and Guatemala Sierras. Mesoamerican *Cypripedium* flowers show remarkable morphological characteristics: the largest flowers are found for taller *C. irapeanum* plants, and the smallest flowers are found for *C. dickinsonianum* (Figure 1a–c). The colors vary from a striking pale-yellow to canary yellow, and the staminode (sterile stamen) is often showy to welcome the insect, which makes its way to a backdoor exit into the pouch. The dorsal sepals are generally not as wide as those in the rest of the cypripediums, and the petals are wider and have slightly smaller synsepals. *C. irapeanum* and *C. molle* also show distinctive sepia-reddish staining inside the lobes, and they have small transparent “windows” over the lip surfaces. The local name of the genus is “pichohuaxtle”, derived from the Náhuatl dialect, which means “bulls’ eggs” [17]. Mexican children crush the showy inflated lip of the flowers for use as whistles or balloons [4,14,18].

**Figure 2:**
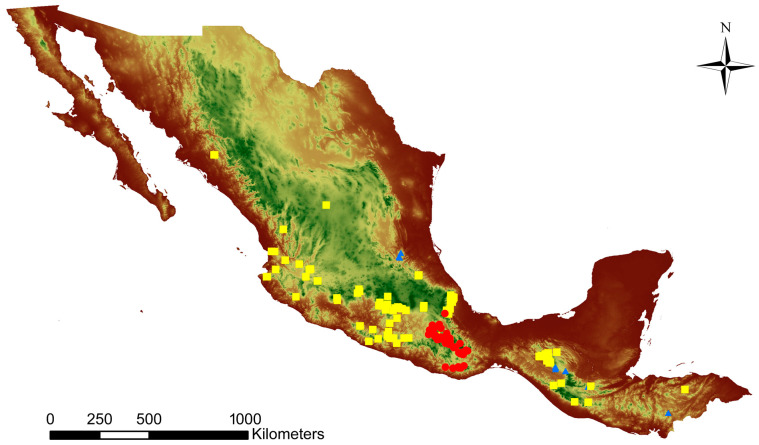
Actual distribution of Mesoamerican *Cypripediums* from herbarium data. *Cypripedium irapeanum* = yellow points, *C. molle* = red points, and *C. dickinsonianum* = blue points.

The Mesoamerican species have dissimilar distributions. *C. dickinsonianum* is restricted to small populations in the Mexican states of Queretaro and Chiapas and in Guatemala and grows on light slopes with *Juniperus*, and is sometimes sympatric with *C. irapeanum* [13,14,18]. In contrast, *C. irapeanum* is widely distributed in shrubs and grasslands or is associated with *Quercus* and *Pinus* in several Mexican states (e.g., Chiapas, Puebla, Mexico, Morelos, Michoacán, Guerrero, Colima, Nayarit, Sinaloa, and Veracruz), and is found in some locations in Guatemala. *Cypripedium molle* is restricted to the Mexican state of Oaxaca and grows in similar forest associations as *C. irapeanum*, but with a difference wherein it develops close to disturbed sites such as roadsides [19] (Figure 2).

## 2. Mesoamerican Slipper Orchids: Unique and Critically Endangered

**Figure 3:**
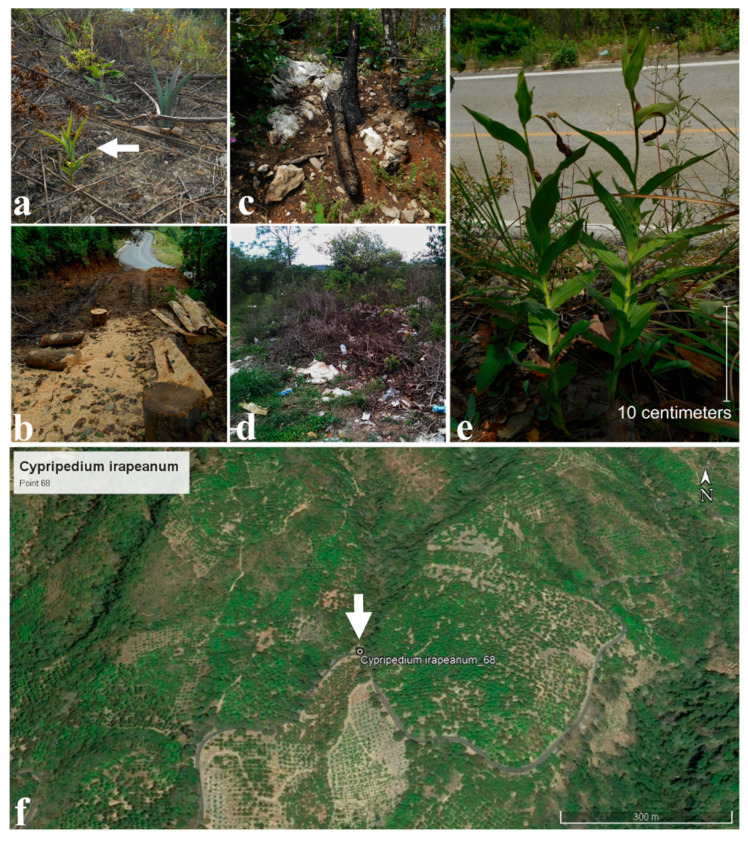
Destroyed habitats of Mesoamerican *Cypripedium molle* in Oaxaca State (**a**–**d**) and *C. irapeanum* in Nayarit State (**e**–**f**). (**a**) Land use change for *Agave angustifolia* cultivation for mezcal production (white arrow indicates a remaining *C. molle* plant). (**b**) Deforestation of a pine forest in the transition from oak to cloud forest habitat. (**c**) Fire destruction of oak forest habitat in limestone. (**d**) Habitat destruction by an outdoor dump. (**e**) Population vulnerability next to the road subject to any perturbation process. (**f**) Land use change of deciduous forest transitions for mango cultivars (white arrow shows the disappeared population). (**a**–**e**), photos taken by Mauricio Moreno in 2019; (**f**), INEGI [23] and Google Earth ©, 2020.

Many factors increase the risk of loss of these species and developing strategies for their conservation is extremely urgent. International treatment CITES (Convention on International Trade in Endangered Species of Wild Fauna and Flora) considers *C. dickinsonianum* as endangered (EN), *C. irapeanum* as vulnerable (VU), and *C. molle* as near threatened (NT) [20]. Mexican policies recognize *C. dickinsonianum* as protected and *C. irapeanum* as EN, while the status of *C. molle* is unconsidered [21]. However, the actual endangered status of all Mesoamerican species is more serious: by using georeferenced entries from Mexican National herbarium records from 1954 to 2013 (176 entries) (Salazar-Chávez, G., personal database), data from previous papers [18], and confirmed records from the website inaturalist.org [22], we obtained 11 records for *C. dickinsonianum*, 102 for *C. irapeanum*, and 57 records for *C. molle*. We determined by using geographic information system techniques, satellite images, georeferenced databases [23], and ecological niche modeling projections [24] that only 3, 43, and 27 of the records, respectively, maintained their original habitats, with a loss of 58% for both *C. dickinsonianum* and *C. irapeanum* and 53% for *C. molle* (Figure 3). Most of the remnants confirmed that populations are endangered due to their proximity to urban sites or sites with probable habitat transformation: from field investigations conducted in 2018 and 2019, we visited 27 recorded *C. molle* populations, and only 3 of them could be found, while the others had been destroyed at the end of 2019 by *Agave angustifolia* plantations used for mezcal production, an alcoholic drink, whose consumption has popularized since the Appellation of Origin was obtained by only the Mexican state of Oaxaca [25]. In the case of *C. irapeanum*, two of five studied populations were subjected to pillage of flowers and plants. Additional threats come from habitat transformation due to road expansion and the establishment of illegal trash dumps. *C. dickinsonianum* is the most vulnerable, and the remaining four populations, which represent half of the total number of records, are near highways and urban centers.

Due to the swift loss of natural habitats for these Mesoamerican species, identification of potential habitats was achieved to identify possible new locations to find or reintroduce populations in the future. Some climatic factors may strongly determine their distributions [18], so ecological preferences and ecological niche modeling have been studied by using the WorldClim-Global Climate Data, which include the temperature seasonality, mean annual precipitation, annual temperature range, mean temperature in the coldest quarter, annual mean precipitation, and precipitation seasonality [26]. We obtained hypothetical projections for the three species by using the maximum entropy algorithm calculated with MAXENT software [27]. To estimate the model, we used herbarium and online records obtained from the website inaturalist.org [22], climatic variables, soil conditions’ layers (e.g., total carbon and nitrogen, bulk density, water-holding capacity, moisture content, and wilting point) [28], altitudinal records, and vegetation types [23] (Table S1), and these data had previously been transformed to compatible formats in ArcGIS© [29].

**Figure 4:**
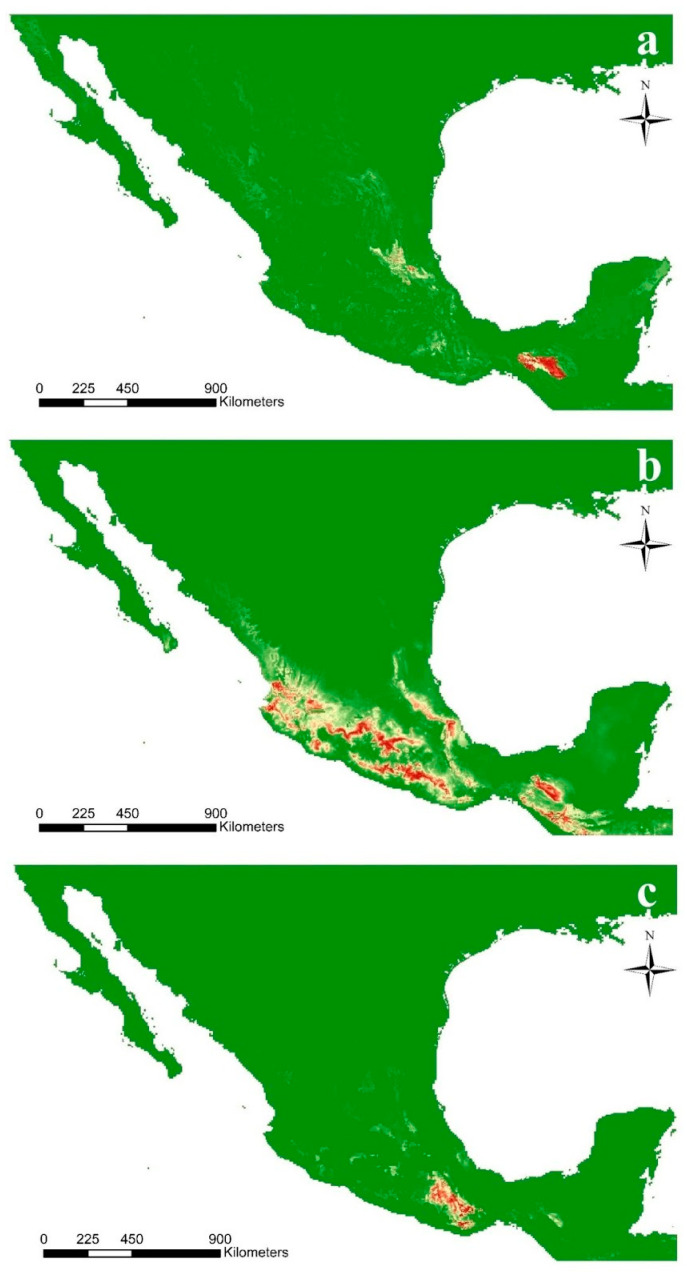
Distribution of suitable habitats of Mesoamerican *Cypripedium* based on the most reliable Maxent model. Rasters generated in Maxent [27]. Maps were generated in ArcGIS© 10.5 (ESRI, https://www.esri.com/en-us/home (accessed on 1 April 2022)) (**a**) *C. dickinsonianum*, (**b**) *C. irapeanum*, and (**c**) *C. molle*.

The resulting projections of the potential habitats for each species indicate that *C. irapeanum* and *C. dickinsonianum* are sympatric in some habitats, whereas certain potential distribution habitats have not yet been recorded (Figure 4a,b). For *C. molle*, the estimated distribution is restricted to the gap located between the union of the eastern and south Sierra Madre located in the physiographic province of Oaxaca´s Sierras Centrales (Figure 4c). The inclusion of soil conditions generates more robust models for suitable habitat projections; however, it is not clear whether soil conditions have a direct impact on plant requirements or the associated microorganisms, such as orchid mycorrhizal fungi (OMF), as we conduct further research [30].

## 3. Underground Growth Pattern in Different Habitats

**Figure 5:**
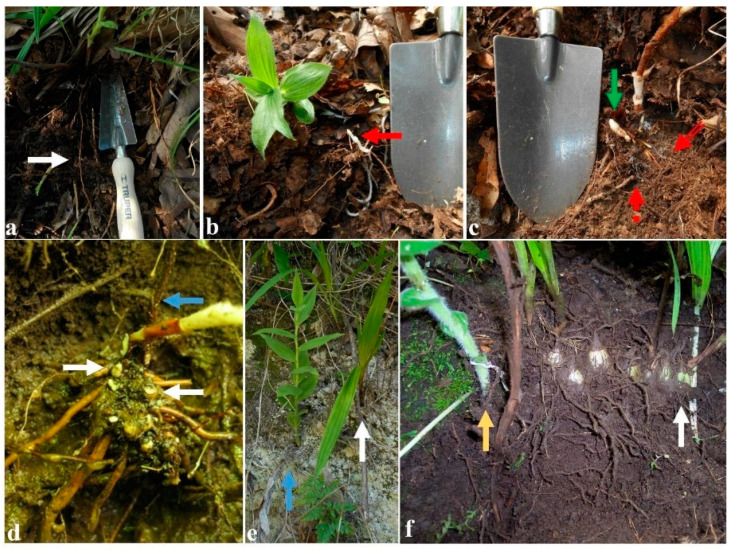
In situ development of *Cypripedium molle* (**a**,**b**) in an oak forest in Oaxaca State and *C. irapeanum* (**c**–**f**) in an oak forest in Mexico State and in a tropical dry forest in Veracruz State. (**a**) Long, little-branched roots longer than 30 cm under abundant litter (white arrow). (**b**) Achlorophyllous shoot (red arrow) of an emerging plant sprouting due to the deep litter where roots develop in the humic horizon. (**c**) Lateral shoot emerging from a near meristematic node (green arrow) and short rhizome development with roots surrounded by mycelial cords (dashed red arrow) with short-distance exploration mycelium (double-line red arrow). (**d**) Multiple points of emergence of annual plants showing a node with growth of several years (white arrows) and the remainder of a dried shoot from the previous year (blue arrow). (**e**) Plants usually associated with *Bletia* orchids (*B. purpurea*, white arrow). (**f**) Association with an eight-year-old *Bletia punctata* plant showing the emerging shoots of the first two submerged bulbs. These belong to the first and second bulbs that developed after germination and the emerging shoots after eight years (white arrow). A dried *C. irapeanum* shoot from the previous year next to a new emerging shoot (yellow arrow). Photos taken by Mauricio Moreno in the year 2019.

**Figure 6:**
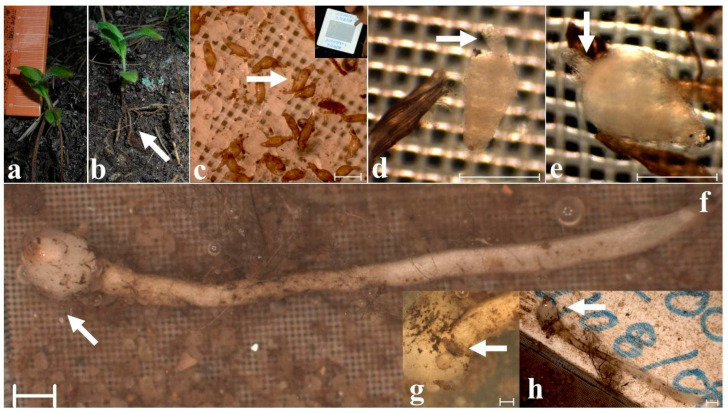
In situ germination of *Cypripedium irapeanum* from Veracruz State in a tropical dry forest (**a**,**b**) and an oak forest in Mexico State (**c**–**h**). (**a**) Seedling less than 3 cm long. (**b**). Emergence of multiple large roots from seedlings (white arrow). (**c**) Seed imbibition (white arrow) after the second month of baiting placement (upper right picture), showing that the sand grain sizes are larger than those of the seeds and brown fungal hyphae (scale bar = 500 µm). (**d**,**e**) Two-month achlorophyllous protocorms in the baiting showing development of the shoot apical apex (scale bar = 500 µm). (**f**) Germinated seed after 10 years of baiting in August 2018 with a root that is several times larger than the undeveloped shoot (white arrow) (scale bar = 16 mm). (**g**) Detail of a root emerging from an undeveloped shoot of the same seedling showing testa remains (white arrow) (scale bar = 500 µm). (**h**) Comparative size of seedlings regarding baiting dispositive (see (**c**)) (scale bar = 2 mm). Photos taken by Mauricio Moreno (**a**,**b**) in 2020; Jesus Colín (**c**) in 2012 and M.P. Ortega (**d**–**h**) in 2018.

We analyzed the underground rhizome growth after conducting the field investigations for three habitats: two for *C. irapeanum* (*Quercus* forest in the states of Mexico, Morelos, and Puebla, and Tropical Deciduous Forest (TDF) in Veracruz State) and one for *C. molle* (*Quercus* forest in Oaxaca State). Both species show consistent patterns of underground root growth that are generally less than 10 cm-deep and are located between an undecomposed litter layer and a layer with high organic matter content that is very similar to the growth of other *Cypripedium* species [4,31] (Figure 5). Mesoamerican species develop short rhizomes and an annual stem with several large roots that can persist for years by storing carbohydrates in the form of starch. For other species in the genus, the root systems may live for nearly 14 years, which allows these plants to remain dormant for long periods without aerial shots until adequate conditions for vegetative growth are met. Many years (e.g., ca. 7–16) are necessary to develop full-grown flowering plants from seedlings of lady slipper orchid species [4,32,33,34]. For *C. irapeanum*, the populations in *Quercus* forest grow under layers rich in organic matter on volcanic-derived acidic soils (pH of 5.6), while populations in TDF grow in poor soils, with high levels of calcium, little organic matter, and a pH of approximately 7.5 [14,35]. Both populations develop under seasonally dry environments. It is worth mentioning that in five years of study on these populations, only one germination event was recorded for each population (Figure 6a,b). The incidence of in situ germination has been estimated to be extremely low (e.g., approximately 0.001%) [8] and can occur over a period of two months (Figure 6c–e) or ten years after seed dispersion (Figure 6f–h). It is not clear whether these scarce germination events depend on the potential of a habitat to provide a germination niche (for example, until colonization by compatible symbiotic fungi) or on the natural long-term dormancy of seeds as a survival mechanism. OMFs are needed for germination and carbon acquisition throughout life to support survival during dormant states, quite common in the genus *Cypripedium* [4,32,36,37,38,39]. In general, the in situ germination requirements for this genus are poorly understood, and long-term studies are needed [40]. Both *C. irapeanum* and *C. molle* grow on different soils, such as reddish, clayey, lateritic, limestone soils or volcanic-derived soils, and this indicates that a broad range of diverse conditions can meet the requirements for germination and increases the potential habitats and probability of establishing new populations [41]. The genus is considered highly dependent on mycorrhizal fungi, and this could be the reason why asymbiotic propagation methods have not been completely established for many species [42,43,44].

## 4. Mycorrhiza Studies for Conservation Purposes

*Cypripedium* species, like other orchids, have two main symbiotic relationships: with insects for pollination and seed production and with OMF for germination and nutrient transfer. For pollination, there are several genera of insects that can function as *Cypripedium* pollinators, and despite the different reports on these insects, few actual successful pollinia removal events have been recorded [45,46,47]. In the case of Mesoamerican *C. irapeanum* and *C. molle*, Halictideae wasps have been considered as pollinators for both, and the only pollinator for *C. irapeanum* is considered as *Lasioglossum nyctere*, while *C. dickinsonianum* has been recognized as self-pollinating [13,14].

**Figure 7:**
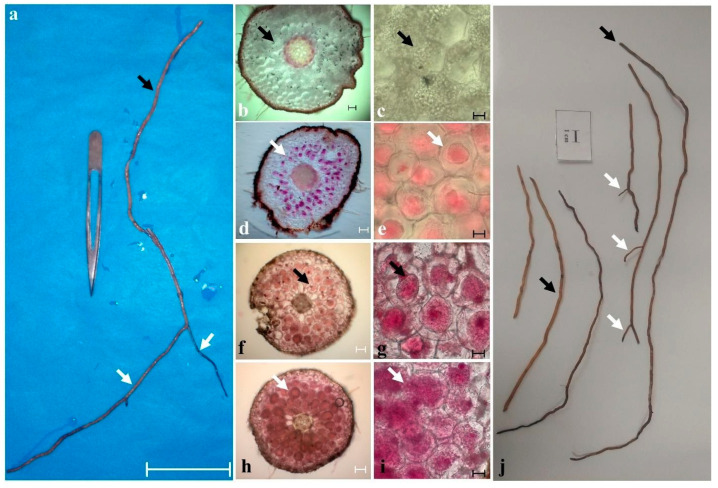
Mycorrhizal root colonization of Mesoamerican *Cypripedium*. View of one of the main roots (black arrows) and secondary roots (white arrows) of *Cypripedium irapeanum* (**a**–**e**) and *C. molle* (**f**–**j**). Cortical colonization by mycorrhizal fungi occurs in patches on the main roots with long sections without fungal colonization and starch grains (**b**,**c**) and some with very dense colonization with digested pelotons (**f**,**g**). SR are always densely colonized with undigested and partially digested pelotons (**d**,**e** and **h**,**i**). Bars in c, e, g, i = 10 µm and bars in b, d, f, h = 100 µm. Bar in a = 10 cm. Photos taken by Mauricio Moreno in year 2019.

However, mycorrhizal symbiotic associations have more effectively studied for several species in adult plants, and scarce and erratic patterns of root colonization [37,48]. We confirm two main patterns of mycorrhiza development for Mesoamerican *C. irapeanum* and *C. molle*: On the main roots, colonization is scarce and can remain for years with highly degraded hyphal coils and large numbers of starch granules [37,48,49]. In contrast, the secondary roots, which are usually short and numerous (ca. 2–3 cm), lack starch reserves because they are actively growing and are highly colonized by hyphal coils in diverse stages of digestion, while most of them are undigested (Figure 7). Seedlings that develop by in situ germination of both species are always colonized with the same pattern as the secondary roots.

**Figure 8:**
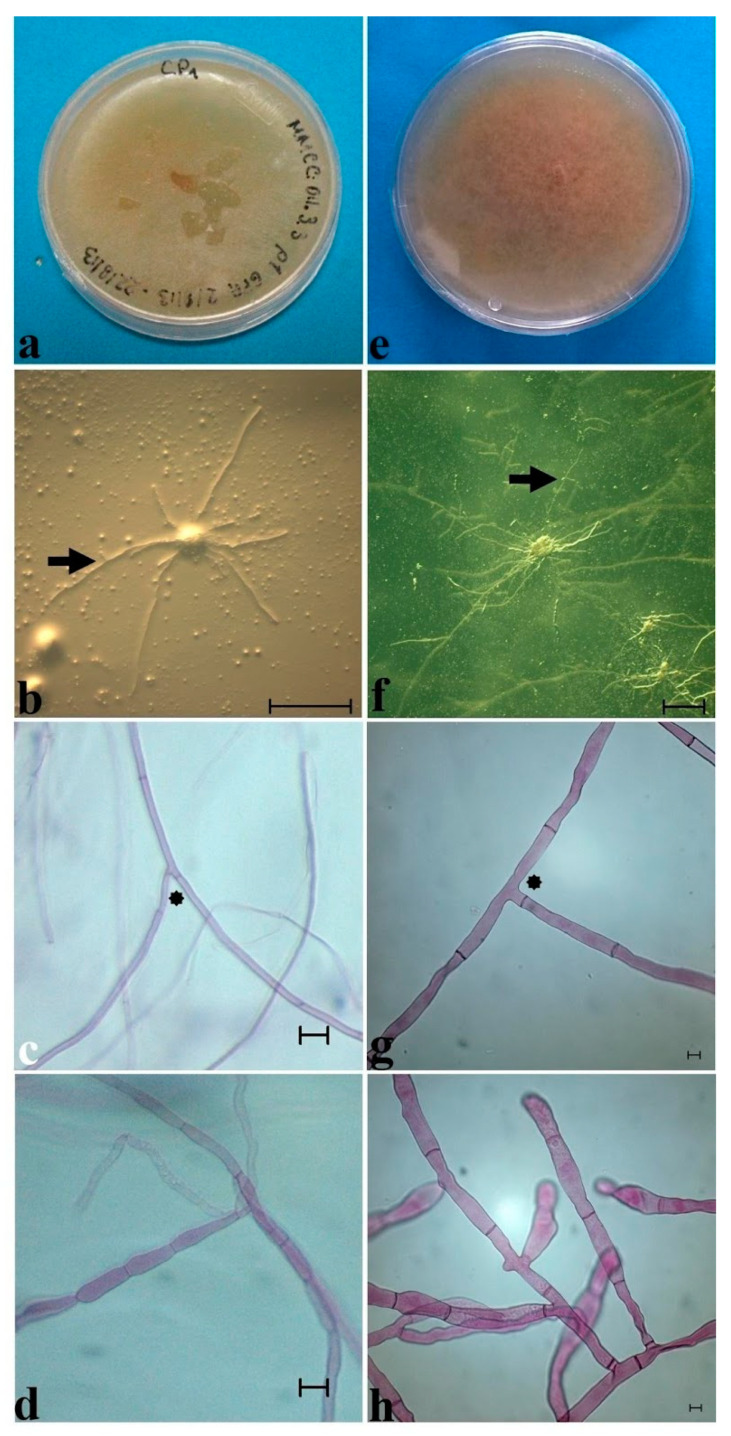
Mycorrhizal isolates of Mesoamerican *Cypripedium*. Isolate from *Cypripedium irapeanum* (**a**–**d**) and from *C. molle* (**e**–**h**). (**a**) Colony view of *Epulorhiza* sp. (*Tulasnella* sp.) from a tropical dry forest in Veracruz State showing a submerged mycelium, yellow-creamy color, and waxy surface. (**b**) Superficial growth after 5 days of incubation of straight hyphae obtained from a peloton after sowing on GPA medium showing 90° bifurcation of the hyphae (black arrow). Bar = 100 µm. (**c**) Mycelium from an isolated culture showing less than 4 µm hyphae and straight basal septa (black asterisk). Bar = 10 µm. (**d**) Monilioid cells. Bar = 10 µm. (**e**) Colony view of *Ceratorhiza* sp. (*Ceratobasidium* sp.) that was isolated from a *Quercus* forest in Oaxaca State showing an aerial mycelium, brownish-creamy color, and cottony superficial aspect. (**f**) View of hyphae growing from a submerged peloton with a knobby appearance after 3 days of incubation. Bar = 10 µm. (**g**) Mycelium from an isolated culture showing hyphae longer than 4 µm and constrained basal septa (black asterisk). Bar = 10 µm. (**h**) Monilioid cells. Bar = 10 µm. Photos taken by Mauricio Moreno in 2020.

There have only been three successful attempts to isolate OMF on *Cypripedium*, for *C. macranthos* var. *rebunense* [43] and Mesoamerican *C. irapeanum* [35] and *C. molle* (Moreno-Camarena and Ortega-Larrocea, submitted). The mycorrhizal endophytes that were obtained from the roots of adult *C. irapeanum* plants from two habitats belong to the anamorphic genus *Epulorhiza* (hyphae less than 4 µm, pearly monilioid cells, creamy submerged colonies on PDA, and slow growth rates ca. 0.2 mm per day). Endophytes that were obtained from *C. molle* show characteristics of the anamorph *Ceratorhiza* (hyphae of more than 4 µm, barrel-shaped monilioid cells, brownish colonies on PDA, aerial mycelium, and growth rates of 0.5 mm/day) (Figure 8, Table 1) [50]. The isolation and long-term conservation of these isolates was achieved only on Green Pea Agar medium [51], AWA (Acidic Water Agar), and FIM (Fungal Isolation Medium) [52]. In media such as PDA (potato dextrose agar) or OMA (oatmeal agar), which are usually used for OMF cultivation, no long-term growth was achieved, and the isolates lost viability in both cases.

Since OMF isolation has been difficult for *Cypripedium* species, using molecular tools to identify the relevant fungi is usually conducted by using the fungal coils of adult roots. The genomic regions studied are the ITS (internal transcribed spacer), LSU (large subunit), and SSU (short subunit) [53,54,55]. Molecular identifications indicate that the genera are sometimes associated with distant phylogenetic fungal groups (Table S2) [56]. In addition, some of the identified fungal partners may be incidental inhabitants, pathogens, or temporal successors [36]. The most common OMFs associated with *Cypripedium* belong to the family Tulasnellaceae: *Tulasnella cystidiophora*, *T. calospora*, and *T. deliquescens*, which were found in 28, 12, and 5 species, respectively. The family Ceratobasidiaceae (*Ceratobasidium cornigerum*) has also been identified in *C. californicum* [37,44,48,57]. However, most of these identifications have been conducted on adult plants, since symbiotic germination is poorly documented and the identification of fungal germination promoters is needed [42,43,58].

**Figure 9:**
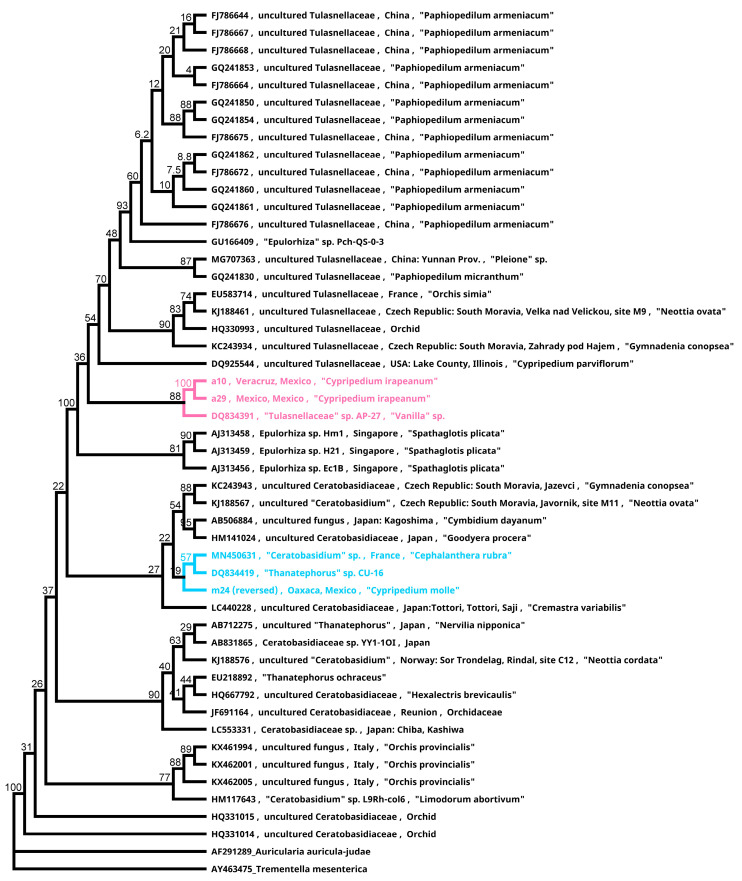
Phylogenetic relationships of endophytes isolated from Mesoamerican *Cypripedium*. The pink clade represents isolates from *Cypripedium irapeanum* from a tropical dry forest and *Quercus* forest. The blue clade contains the isolate from *C. molle*, which was inferred by using the maximum likelihood method and the Tamura-Nei model log likelihood of –3241.26. The branch length labels represent the bootstrap proportions. Analyses were conducted in Geneious (2021.0.3).

To analyze the relationships among fungi that were isolated from the adult roots of *C. irapeanum* (*Quercus* forest in Mexico State and TDF in Veracruz State) and plantlets of *C. molle* (*Quercus* forest in Oaxaca State), we amplified their ITS regions by using the primer combination ITS 1/ITS 4 [36,37,48,51,57,59], and the resulting sequences were assembled and edited with Geneious (2021.0.3). The sequences are deposited in the GenBank-NCBI database. A search for the most similar sequences was conducted by using the BLAST algorithm [60,61], and alignments were conducted using MAFFT [62]. Both algorithms are contained in the Geneious software. Phylogenetic reconstruction was carried out using PHYML plugin [60,63] by using the maximum likelihood method and the Tamura-Nei model with bootstrap support of 1000 replicates (Figure 9).

Significant results were observed from the phylogenetic reconstruction. The isolates from *C. molle* plantlets belong to Ceratobasidiaceae in a clade that consists of terrestrial endophytes of *Vanilla* spp. from Puerto Rico and *Cephalanthera rubra* from France [64] (Figure 9). Conversely, the isolates of the widespread *C. irapeanum* were recovered from different habitats (e.g., *Quercus* forest and TDF) and belong to Tulasnellaceae in a clade that includes a terrestrial endophyte from *Vanilla* [50], which indicates a probable high level of specificity to a widely distributed soil generalist fungal clade (Figure 9). This behavior is opposite to that of the North American *C. californicum*, a soil specialist (always grows on serpentine soils) but is associated with various species of the genus *Tulasnella* [48]. The same is true for the terrestrial *Dichromanthus* that form specific associations with a particular mycorrhizal endophyte when the species are widely distributed in several habitats and soil conditions. However, in one restricted habitat, the species were associated with several clades of the same fungal family (López-Reyes and Ortega-Larrocea, personal communication).

The fact that plantlets of *C. molle* associate with Ceratobasidiaceae may indicate that the fungi from this family are germination promoters, as was proposed for *C. calceolus* [65]. This could explain why endophytes isolated from adult plants do not promote good germination, as has been observed in *C. irapeanum* [35] and previously in *C. macranthos* var. *rebunense* [51]. The isolation and identification of fungi from other populations and plant stages together with cultivation by asymbiotic and symbiotic methods would provide a better understanding of germination, specificity, fungal succession during plant lifetimes, distribution of populations, and microhabitat requirements to develop conservation strategies for these unique species.

## 5. Symbiotic and Asymbiotic Germination and Development

Seeds of *Cypripedium* spp. have tests that consist of a hydrophobic double-layer rich in lignin that allows them to float on water [66,67]. At maturity, these seeds have high concentrations of abscisic acid (ABA) that can delay germination [68,69] which are probably involved in long-term survival through the years and persist for at least four years to form seed banks in soils [4,42,70]. In situ germination relies on mycorrhizal fungi that can penetrate the testa and induce germination [42,43]. Symbiotic germination under in situ conditions is poorly studied in *Cypripedium* and probably occurs in spring or early summer at 2–5 cm soil depths in moisture-stable sites within a pH range from 5.3 to 8.1 [31]. In some species of the genus, germination is stimulated by cold (e.g., *C. calceolus*, *C. lentiginosum*, *C. macranthos* var. *rebunense*) because they grow in temperate regions [42,43,68,71].

**Figure 10:**
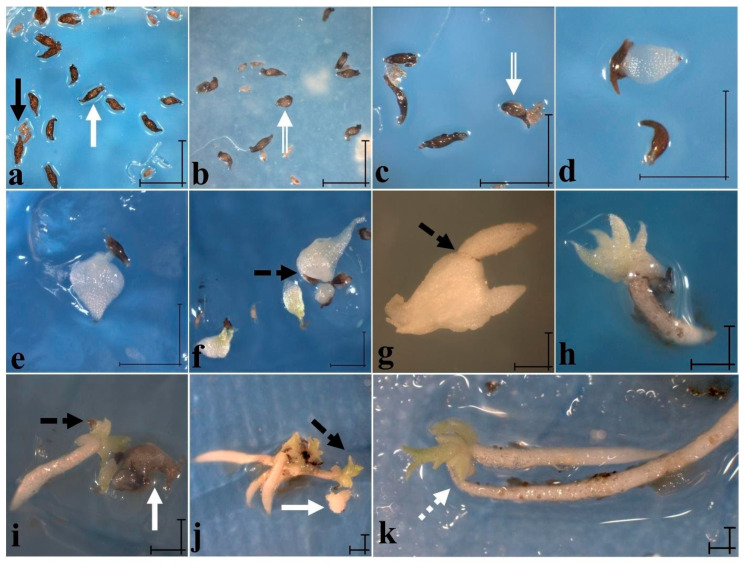
Asymbiotic in vitro germination of *Cypripedium irapeanum* in Norstog medium. (**a**) Development stage 0 on day of sowing (black arrows show immature and undeveloped seeds and white arrows show mature seeds). (**b**) Imbibed seeds two days after sowing (das) (white arrow) (development stage 1). (**c**) Imbibed seed with rupture of testa 12 das (white arrow) (development stage 2). (**d**) Protocorm polarization with apical meristem at 45 das (upper structure) (developmental stage 3). (**e**) Protocorm development with leaf blade at 54 das (developmental stage 4). (**f**) Pre-seedling showing radical meristem (black dashed arrow) and a long achlorophyllous leaf 96 das (developmental stage 5). Other chlorophyll pre-seedlings with two leaf blades are located at the bottom left. (**g**) Seedling development with two incipient roots at 122 das (black dashed arrow) (developmental stage 6). (**h**) Seedling 187 das with four leaves and a greater ratio between root and shoot. (**i**) Seedling 300 das with lateral shoots and remnant testa (black dashed arrow) from the first shoot and two radical sprouts. The white arrow shows two oxidized pre-seedlings at stage 4. (**j**) Four seedlings 300 das with long roots and one seedling with two shoots (black dashed arrow). The white arrow shows a protocorm with irregular protuberances, such as rhizoids, or a protocorm-like body (PLB). (**k**) Seedling at 365 das with three shoots and two elongated and exfoliant rhizodermic roots. Scale bars = 100 µm.

In vitro germination of Mesoamerican species has been studied using symbiotic and asymbiotic approaches. Asymbiotic germination was tested on Phytamax^TM^ (SIGMA) with sucrose 2% [72], Murashige and Skoog [73], Norstog [74], and oatmeal agar [52] at pH 5.6 after the seeds were stimulated by cold storage (4 °C) for four months to break dormancy (Moreno-Camarena and Ortega-Larrocea, in process). However, seedlings developed only on Norstog medium (Figure 10). It is worth mentioning that while Asian or European *Cypripedium* species germinate at 4 to 54 months after sowing [42,58], *C. irapeanum* can germinate at 12–14 days after sowing (das) [35]. Germination development, in vitro or in situ, begins when the testa splits and the embryo swells, which gives rise to a protocorm with a promeristematic zone (Figure 10a–c). From this, the apex begins to grow, which is followed by root formation on the opposite point of the protocorm (Figure 10d–f). The protocorms are greenish and usually have long rhizoids [31,42], except for *C*. *irapeanum*, which does not develop rhizoids under asymbiotic in vitro conditions (Figure 10d–g). Roots emerge after shoot differentiation of two foliar sheets (Figure 10g). This root system continues to grow faster over the shoot leaves and develops into several small sheets and lateral shoots (Figure 10h–k).

**Figure 11:**
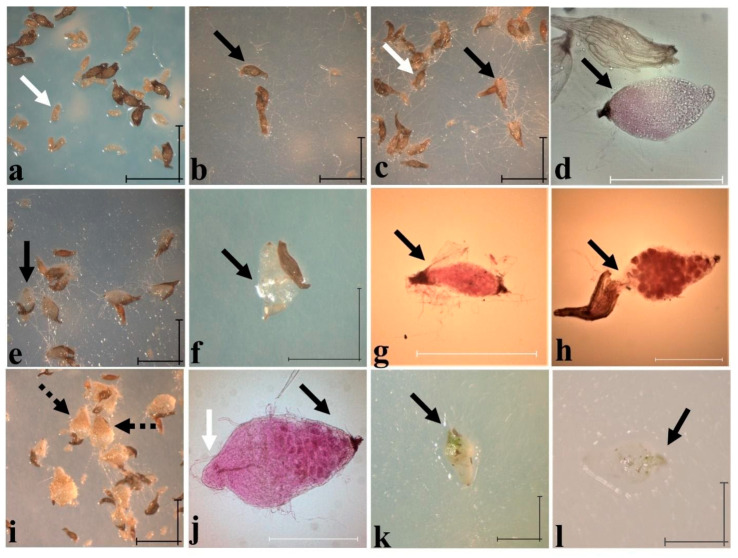
Symbiotic in vitro germination of *Cypripedium irapeanum* with two *Epulorhiza* spp. (*Tulasnella* spp.) isolates obtained from an oak forest in Mexico State and a tropical deciduous forest in Veracruz State with seeds from Puebla State. (**a**) Seed imbibition (stage 1) before fungal contact at 2 days after sowing (das) (white arrow shows immature and ungerminated seeds with embryos) with a Mexico State isolate. (**b**) Mycorrhizal contact during seed imbibition at 12 das with the Veracruz isolate. (**c**) Symbiotic protocorms surrounded by mycelium (not rhizoids) at stages 2 (white arrow) and 3 developing rhizoids (black arrow) at 12 das with a Veracruz isolate. (**d**) Protocorm out of testa at 12 das stained with acid fuchsine showing pelotons at the micropillar seed region (black arrow) and hyphae growing in medium. (**e**,**f**) Symbiotic protocorms at stages 3 (**e**) and 4 (**f**) at 22 and 54 das, respectively, with the Veracruz isolate showing early vitrification and fungal incompatibility (black arrows). (**g**,**h**) Histological staining evidence of the fungal incompatibility, where pelotons invade apical meristematic tissue cells (black arrows) at stage 3 (**g**) and stage 4 (**h**), both at 109 das with an isolate from Mexico State. (**i**) Symbiotic protocorms at 187 das showing fungal compatibility that are located in a brownish tissue in the micropylar zone (black dashed arrow) with the Veracruz isolate. (**j**) Histological view of a stained mycorrhized protocorm with dense pink pelotons (black arrow) at the micropylar pole and with intact leaf vascular bundles (white arrow) at 96 das. (**k**,**l**) Symbiotic mixotrophic protocorms at stage 4 (with two foliar sheets) at 72 and 109 das with the Veracruz isolate. Scale bar = 100 µm. Photos taken by Mauricio Moreno in 2015.

Symbiotic germination was tested on *C. irapeanum* seeds that were obtained from a population located in an oak forest in Puebla State. Two mycorrhizal endophytes (e.g., *Epulorhiza* spp.) that were isolated from adult plants from two different sites (e.g., a *Quercus* forest from Mexico State and a TDF from Veracruz State) were tested, and they exhibited 35% and 20% germination rates, respectively (Table 2). While the isolate from Mexico State promoted a higher germination percentage, the *Epulorhiza* isolate from Veracruz promoted a more compatible and advanced developmental stage (Figure 11). Shimura and Koda [43] state that symbiotic germination begins after some weeks of incubation in *C. macranthos* var. *rebunense* after cool storage, at which time the embryo is imbibed and develops rhizoids that serve as the entry points for fungal colonization. In the case of *C. irapeanum*, rhizoids did not develop under either asymbiotic or symbiotic conditions (Figure 11a–f), with mycorrhizal colonization likely beginning from the micropylar end (Figure 11d). This colonization is also suitable for water and nutrient absorption [75,76], even if suspensor cells are degraded in the *C. calceolus* and Mesoamerican hybrid *Cypripedium* × *fred-mulleri* to form spherical embryos [18,67]. However, neither of the two identified isolates allow protocorms to develop seedlings (stage 6), as in asymbiotic germination (Figure 10). This incompatibility after symbiotic germination is observed when complete cells that form the protocorm are invaded by fungi, which prevents the apical meristematic cells from undergoing division and growth (Figure 11f–h). This phenomenon could be due to the origin of the isolates, since all were obtained from adult plants [51] or because *Cypripedium* compatibility with mycorrhizal fungi is low under natural conditions and resulted in the low recruitment observed. Shimura and Koda [43] achieved seedling formation with shoots after cultivation of symbiotic germinated protocorms in an antifungal medium, which indicated that autotrophic plants cannot regulate the symbiotic balance. Other attempts to obtain symbiotic plants have been reported and were without success [65,77].

By comparing asymbiotic and symbiotic development under in vitro conditions, imbibition, rupture of the testa, and tissue differentiation (polarization) occur at similar times after sowing. The multiple lateral shoot induction observed in asymbiotic seedlings produced in vitro could be a consequence of growth under an artificial environment because in situ seedlings developed only one shoot. Unfortunately, there is not yet a successful protocol for propagating lady slipper orchids under either approach.

## 6. Final Remarks

Mesoamerican *Cypripedium* species represent an interesting group for study due to their phylogenetic importance as a sister ancestral group to the rest of the genus and because all *Cypripedium* species are endangered and have some degree of importance. As seen here, their mycorrhizal preferences differ from other species in the genus, and it is probable that they have ecological preferences and, consequently, their morphological variations could be derived from these. The high endangered status of the remaining populations prompts the generation of information on the OMF that promotes in situ germination to develop priority mechanisms for the conservation of hotspot habitats.

Since orchids depend on symbiotic associations, conservation of these species depends on an understanding of the biological and ecological factors that drive the distributions of mainly fungal partners [78,79]. Some aspects may be more determinant than others in species establishment, such as the distributions of suitable mycorrhizal germination-promoting fungi [80]. This contribution provides a partial view of some aspects of the biology of Mesoamerican *Cypripedium*; however, the successful pollinators, in situ symbiotic germination, and OMF specificity remain unknown. The destruction of habitats urges ex situ conservation strategies that must include not only seed collections but also mycorrhizal fungi that can promote compatible germination or facilitate adaptation. Clarifying the structures of the mycorrhizal fungal communities and isolating germination-promoting fungi would help to determine suitable habitats to conserve or re-establish the studied species and the viability and persistence of in situ seed banks. Asymbiotic propagation in suitable media is a promising tool, but little is known about asymbiotic plant ex vitro adaptation. Both approaches would ensure long-term conservation of these highly endangered species before most of their habitats disappear.

## Figures and Tables

**Table 1:** Micromorphological characteristics of *Cypripedium* spp. Mycorrhizal endophytes (mean ± standard deviation). Different letters represent significant differences at *p* ≤ 0.05. See Figure 8.

Plant Species	Mean Day Growth on PDA Medium	Basal Septa (µm)	Diameter of Hyphae (µm)	Monilioid Cells (µm)	
			Mean ± SD	Width	Length
*Cypripedium irapeanum* (Tropical deciduous forest, Veracruz)	1.14 ± 0.22a	1.794 ± 1.280a	3.3 ± 1.1a	7.7 ± 2.0a	16.1 ± 4.5a
*C. irapeanum* (*Quercus* forest, Mexico State)	0.44 ± 0.25b	1.439 ± 0.384a	2.7 ± 0.4a	7.8 ± 1.2a	18.5 ± 4.2a
*C. molle* (*Quercus* forest, Oaxaca)	2.50 ± 1.20c	7.368 ± 1.679b	12.6 ± 1.2b	20.0 ± 1.9b	36.2 ± 8.4b

**Table 2:** Asymbiotic (1–4 treatments) and symbiotic (5–6 treatments) germination and development of *Cypripedium irapeanum* seeds after 256 days of sowing. MS = Murashige and Skoog [73], Modified Phytamax ™ [72], Norstog [59], OMA = Oatmeal agar [52]. Stages can be appreciated in Figure 8 and Figure 9.

Treatments	STAGES (%)					
	E0	E1	E2–3	E4	E5	E6
	Immature and Ungerminated Seeds with Embryos	Imbibition	Rupture of the Testa and Polarization of the Embryo	Foliar Elongation	Root Meristematic Differentiation	Seedling
MS	49	99	0	1	0	0
Phytamax ™	71	88	10	0	2	0
Norstog	52	91	1	3	1	4
OMA	49	86	14	0	0	0
TDF isolate	29	81	19	1	0	0
*Quercus* forest isolate	26	65	24	11	0	0

## Data Availability

The data presented in this study are available in the text and supplemental data. The data presented in this study are available on request from the corresponding author.

## Supplementary Materials

The following supporting information can be downloaded at: https://www.mdpi.com/article/10.3390/plants11121554/s1, Supplementary Table S1. Habitat characteristics of *Cypripedium* spp. remnant populations used for projection of niche ecological model. Supplementary Table S2. Some studies in *Cypripedium* endophytes using metabarcoding or traditional approaches, entries are ordered in chronological descendent order.
