# Supplementary material for: Mesoamerican Cypripedium: Mycorrhizal Contributions to Promote Their Conservation as Critically Endangered Species
Source: Plants (Basel). 2022 Jun 12;11(12):1554. doi: 10.3390/plants11121554 (PMC9227847; doi:10.3390/plants11121554)
Supplement: Supplementary file 1 [file plants-11-01554-s001.zip › plants-1715746-supplementary.pdf]

Table S1. Habitat characteristics of *Cypripedium* spp. remnant populations used for proyection of niche ecological model.

Species	Vegetation	Precipitation (mm)	Soil Type	Clime Code	Clime Type	Altitude (mosl)
Cypripedium dickinsonianum	CF	1200–1500	Lithosol	(A)C(m)	TH	1517
	OPF	1500–2000	Luvisol	C(m)(w)	TH	1400
	OPF	800–1200	Luvisol	(A)C(w1)(w)	TSH	1527
Cypripedium irapeanum	OPF	1200–1500	Luvisol	C(w2)(w)	TSH	1797
	OPF	1200–1500	Luvisol	C(w2)(w)	TSH	1782
	OPF	1200–1500	Luvisol	C(w2)(w)	TSH	2196
	OPF	1200–1500	Regosol	A(C)m(w)	TH	1574
	OPF	1500–2000	Andisol	(A)C(m)(w)	TH	1702
	TDF	1200–1500	Rendzina	A(C)w1(w)	TSH	1822
	OPF	600–800		C(w2)(w)	TSH	2570
	TDF	800–1200	Vertisol	C(w0)(w)	TSH	2047
	OPF	1200–1500	Luvisol	C(w2)(w)	TSH	1606
	OPF	1200–1500	Luvisol	C(w2)(w)	TSH	1867
	OPF	1200–1500	Luvisol	C(w2)(w)	TSH	2033
	OPF	1500–2000	Luvisol	(A)C(m)	TH	1409
	OPF	1200–1500	Andisol	C(w2)(w)	TSH	2158
	OPF	800–1200	Lithosol	(A)C(w2)(w)	TSH	1574
	OPF	1500–2000	Cambisol	Aw2	WS	885
	OPF	1200–1500	Lithosol	(A)C(w2)(w)	TSH	1574
	OPF	1200–1500	Andisol	C(w2)(w)	TSH	2074
	TDF	800–1200	Regosol	Aw0	WS	921
	OPF	1500–2000	Cambisol	(A)C(w2)(w)	TSH	1692
	OPF	1200–1500	Andisol	A(C)w2(w)	TSH	1744
	OPF	1200–1500	Andisol	A(C)w2(w)	TSH	1812
	OPF	800–1200	Cambisol	A(C)w2(w)	TSH	1456
	TDF	1200–1500	Feozem	Aw1(w)	WS	325
	TDF	600–800	Feozem	(A)C(w0)(w)	TSH	1634
	TDF	800–1200	Regosol	(A)C(w1)(w)	TSH	1508
	TDF	600–800	Vertisol	(A)C(w0)(w)	TSH	1584
	TDF	800–1200	Lithosol	Aw0(w)	WS	881
	OPF	600–800	Luvisol	C(w2)(w)	TSH	2301
	TDF	800–1200	Acrisol	Aw0(w)	WS	1247
	TDF	600–800	Vertisol	C(w0)(w)	TSH	1909
	TDF	600–800	Vertisol	C(w2)(w)	TSH	1965
	TDF	800–1200	Feozem	Aw0(w)	WS	1324
	OPF	1200–1500	Acrisol	(A)C(w2)(w)	TSH	1055
	OPF	800–1200	Feozem	C(w1)(w)	TSH	2099
	TDF	800–1200	Lithosol	C(w2)	TSH	1226
	CF	1200–1500	Luvisol	Aw1(w)	WS	609
	CF	1500–2000	Acrisol	C(m)(w)	TH	1316
	OPF	1500–2000	Feozem	C(m)	TH	1762
	OPF	1500–2000	Acrisol	(A)C(m)	TH	1201
	CF	1500–2000	Luvisol	Aw2(w)	WS	582
	CF	1500–2000	Luvisol	(A)C(m)	TH	984
	CF	1500–2000	Luvisol	(A)C(m)	TH	876

	CF	1500–2000	Luvisol	(A)C(m)	TH	1089
	TDF	600–800	Cambisol	A(C)w0(w)	TSH	1786
	CF	1200–1500	Luvisol	Aw1(w)	WS	552
	CF	1200–1500	Rendzina	(A)C(m)	TH	1026
	TDF	800–1200	Regosol	Aw0(w)	WS	680
	TDF	800–1200	Lithosol	A(C)w1(w)	TSH	1636
	OPF	1200–1500	Andisol	A(C)w2(w)	TSH	1744
	OPF	800–1200	Feozem	C(w1)(w)	TSH	2061
Cypripedium molle	TDF	600–800	Feozem	BS1hw(w)	SDSH	1690
	OPF	800–1200	Regosol	C(w0)(w)	TSH	1804
	OPF	2500–4000	Regosol	C(w2)(w)	TSH	1785
	TDF	800–1200	Cambisol	C(w0)(w)	TSH	2081
	TDF	800–1200	Cambisol	C(w0)(w)	TSH	2217
	OPF	600–800	Lithosol	C(w1)(w)	TSH	2208
	OPF	600–800	Rendzina	BS1hw(w)	SDSH	1886
	TDF	600–800	Cambisol	A(C)w0(w)	TSH	1872
	OPF	600–800	Lithosol	C(w0)(w)	TSH	2213
	OPF	600–800	Lithosol	C(w0)(w)	TSH	2213
	OPF	1200–1500	Acrisol	C(w2)(w)	TSH	2488
	TDF	800–1200	Lithosol	C(w0)(w)	TSH	2067
	OPF	800–1200	Acrisol	C(w1)(w)	TSH	2115
	OPF	800–1200	Luvisol	(A)C(w0)(w)	TSH	1857
	OPF	800–1200	Luvisol	(A)C(w0)(w)	TSH	1857
	TDF	600–800	Regosol	BS1hw(w)	SDSH	1590
	TDF	600–800	Lithosol	C(w0)(w)	TSH	2018
	TDF	600–800	Cambisol	A(C)w0(w)	TSH	1859
	OPF	800–1200	Cambisol	C(w2)(w)	TSH	2013
	XS	400–600	Regosol	BS1kw(w)	SDT	2065
	TDF	600–800	Cambisol	A(C)w0(w)	TSH	1863
	OPF	400–600	Lithosol	BS1kw(w)	SDT	2190
	OPF	800–1200	Regosol	C(w2)(w)	TSH	2549
	TDF	800–1200	Regosol	A(C)w0(w)	TSH	2041
	TDF	600–800	Regosol	A(C)w0(w)	TSH	1697
	OPF	600–800	Lithosol	C(w2)(w)	TSH	2295
	XS	800–1200	Regosol	C(w1)(w)	TSH	2457
	TDF	600–800	Cambisol	A(C)w0(w)	TSH	1859

CD = Cloud forest, OPF = Oak-pine Forest, TDF = Tropical Deciduous Forest, XS = Xerophilous scrub. TH = Tempered humid; TSH= Tempered subhumid, WS = Warm subhumid, SDSH = Semi-dry semi-humid, SDT = Semidry tempered.

Table S2. Some studies in *Cypripedium* endophytes using metabarcoding or traditional approaches, entries are ordered in chronological descendent order.

Reference	Host orchid	Primers	Identified order/families
Chen et al., 2019	*Cypripedium guttatum*	ITS3/ITS4OF	Sebacinales<br>Tulasnellaceae<br>Ceratobasidiaceae
Farkas-Lasich, 2018	*Cypripedium arietinum*	ITS1F/ITS4	*Phialocephalafortinii*<br>*Leptodontidiumorchidicola*
Oja et al., 2015	*Cypripedium calceolus*<br>*Neottia ovata*<br>*Orchis militaris*	ITS1Fngs/ITS4ngs	Tulasnellaceae-Sebacinales<br>Ceratobasidiaceae<br>Tulasnellaceae
Bunch et al., 2013	*Cypripedium acaule*	ITS1F–ITS4<br>ITS1OF-ITS4OF	*Russulalaccata*<br>*Tulasnellatomaculum*<br>*Cadophorafinlandica*<br>*Cistellaspicicola*<br>*Cladophialophorachaetospira*<br>*Diaporthephaseolorum*<br>*Lactariusimperceptus*<br>*Meliniomycesvariabilis*<br>*Oidiodendronmaius*

			Phialeastrobilina<br>*Phialocephalafortinii*<br>*R. virescens*<br>*Sorocyberesinae*<br>*T. asymmetrica*<br>*T. pruinosa*
Whitridge, 2004	*Cypripedium fascicula-tum*	ITS1F/ITS4	*Russula*<br>*Lactarius*<br>*Suillus*<br>*Tulasnella*
